# Application of protection motivation theory in epidemic prevention in patients with respiratory diseases under the COVID‐19 pandemic: A cross‐sectional study

**DOI:** 10.1111/crj.13693

**Published:** 2023-09-04

**Authors:** Jian Guan, Yingying Zhang, Shan You, Yujing Li, Hongxing Zhao, Weiqin Bu, Yanping Xie

**Affiliations:** ^1^ Department of Respiration, the First People's Hospital of Huzhou the First Affiliated Hospital of Huzhou University Huzhou China; ^2^ XuanCheng Vocational & Technical College Xuancheng China; ^3^ School of Nursing Huzhou University Huzhou China; ^4^ Department of Nursing, the First People's Hospital of Huzhou the First Affiliated Hospital of Huzhou University Huzhou China; ^5^ Department of Radiology, the First People's Hospital of Huzhou the First Affiliated Hospital of Huzhou University Huzhou China; ^6^ Department of Endoscopy Center, the First People's Hospital of Huzhou the First Affiliated Hospital of Huzhou University Huzhou China

**Keywords:** COVID‐19, protection motivation theory, respiratory diseases, self‐efficacy, self‐management behavior

## Abstract

**Objective:**

This study aimed to investigate the effects of nursing intervention based on protection motivation theory (PMT) on patients with respiratory diseases in the context of the Coronavirus Disease 2019 (COVID‐19) pandemic.

**Methods:**

A total of 74 patients with respiratory diseases who were hospitalized from June 2020 to February 2021 were enrolled and stratified into a control group (*n* = 37) and an experimental group (*n* = 37) according to a stratified random sampling method. The control group adopted a routine nursing intervention program of the respiratory department, whereas the experimental group received a PMT‐based nursing intervention program on the basis of the control group. Chronic Disease Self‐Management Study Measures (CDSMS) and Self‐Efficacy for Managing Chronic Diseases 6‐item Scale (SECD6) were used to evaluate the effect of PMT intervention before intervention, after 1 week, and after 4 weeks of intervention. The levels of forced vital capacity (FVC), forced expiratory volume in 1 second (FEV1), FEV1/FVC and peak expiratory flow (PEF) were measured to evaluate pulmonary function.

**Results:**

Before the intervention, there were no significant differences in the scores of CDSMS and SECD6 scales and liver function indexes between the two groups (*p* > 0.05). After 1 and 4 weeks of intervention, the scores of CDSMS and SECD6 scales of the experimental group were significantly higher than those of the control group (*p* < 0.0001). The indexes of pulmonary function of the experimental group were improved, but there was no significant difference compared with the control group (*p* > 0.05).

**Conclusion:**

Nursing intervention based on PMT contributes to the improvement of self‐management behaviors and self‐efficacy, which is conducive to the prognoses of patients.

## Graphical abstract

The effect of PMT‐based nursing intervention was evaluated using CDSMS and SECD6 scales. The results showed significant improvement of the self‐management ability and self‐efficacy of patients with chronic respiratory diseases, which in turn facilitated the recovery of patients' pulmonary function. This study is the first to apply PMT into the implementation of a clear nursing intervention program in patients with chronic respiratory disease during the COVID‐19 pandemic and has achieved satisfactory results.

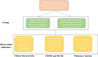

## 1 INTRODUCTION

Since being first reported in Wuhan, China in December 2019, Coronavirus Disease 2019 (COVID‐19) has spread globally.^1^ On 11 March 2020, the World Health Organization declared the COVID‐19 outbreak a global pandemic.^2^ As of 4 September 2022, more than 600 million confirmed cases of COVID‐19 have been reported throughout the world, resulting in more than 6.4 million deaths (http://www.who.org/). In addition to age ≥65 years, which was the strongest risk factor for severe COVID‐19 infection, several comorbidities including malignancy and chronic obstructive pulmonary disease (COPD) were also associated with poor prognoses of COVID‐19 patients.^3^, ^4^ However, during the COVID‐19 pandemic, more attention has been focused on the risk of cardiovascular diseases and less on chronic respiratory diseases, though preexisting respiratory diseases, including asthma, interstitial pneumonia, bronchiectasis and COPD, may exacerbate COVID‐19.^5^, ^6^, ^7^ Under this situation, the clinical nursing of patients suffering from respiratory diseases is facing great challenges. It is worth pondering what appropriate and effective nursing interventions should be taken to improve the physical function of patients with chronic respiratory diseases and therefore reduce the risk of COVID‐19.

Among various theories used to guide behavioral research, protection motivation theory (PMT) has been widely adopted to influence healthy behaviors. PMT refers to the process of explaining behavioral changes by threat appraisal and coping appraisal of the cognitive regulatory process from the perspective of motivation. Threat appraisal includes perceived severity and perceived vulnerability whereas coping appraisal consists of perceived self‐efficacy, response efficacy and cost efficacy of adopting protective behaviors.^8^, ^9^ PMT‐based nursing interventions integrate threat and coping appraisal processes to foster protection motivation. To date, research on PMT behavioral interventions has evolved into a health‐promoting approach and has been employed to prevent multiple public health problems, including oral health care,^10^ and type 2 diabetes.^11^ Clinical studies have shown that PMT threat and coping appraisals are predictors of protective motivation for hospital staff to implement COVID‐19 prevention behaviors.^12^ Results of another cross‐sectional study showed that response efficacy and self‐efficacy positively predicted protective behaviors against COVID‐19, with self‐efficacy playing a greater role.^13^ From this point of view, formulating precise nursing intervention strategies based on PMT to improve threat appraisal and self‐efficacy can facilitate ideal protection motivation and have a good effect in preventing the transmission of COVID‐19 infection. However, the role of PMT in the healthy behaviors of patients with chronic non‐infectious respiratory diseases during the COVID‐19 pandemic remains unclear. This study aimed to explore the effects of PMT‐based nursing intervention on self‐management behaviors and self‐efficacy of patients with chronic non‐infectious respiratory diseases during the COVID‐19 pandemic, to provide a reference for the formulation of effective intervention measures.

## 2 SUBJECTS AND METHODS

### 2.1 Subjects

Sample size estimation: The required sample size was calculated by comparing two sample means: *N* = 2(u_α_ + u_β_)^2^σ^2^/δ^2^.^14^ The test level was set as two‐sided α = 0.05, the effective size was 1.39, and the test efficiency was 1‐β = 0.9. According to relevant research results, δ was set as 4.69, and σ was set as 3.85. u_α_ was set at 1.96, u_β_ was set at 1.28, and the sample size was calculated as *N* = 32. However, considering the 15% loss to follow‐up rate, the number of cases in the experimental group and the control group was finally determined as 37, and the total sample size was 74.

**FIGURE 1:**
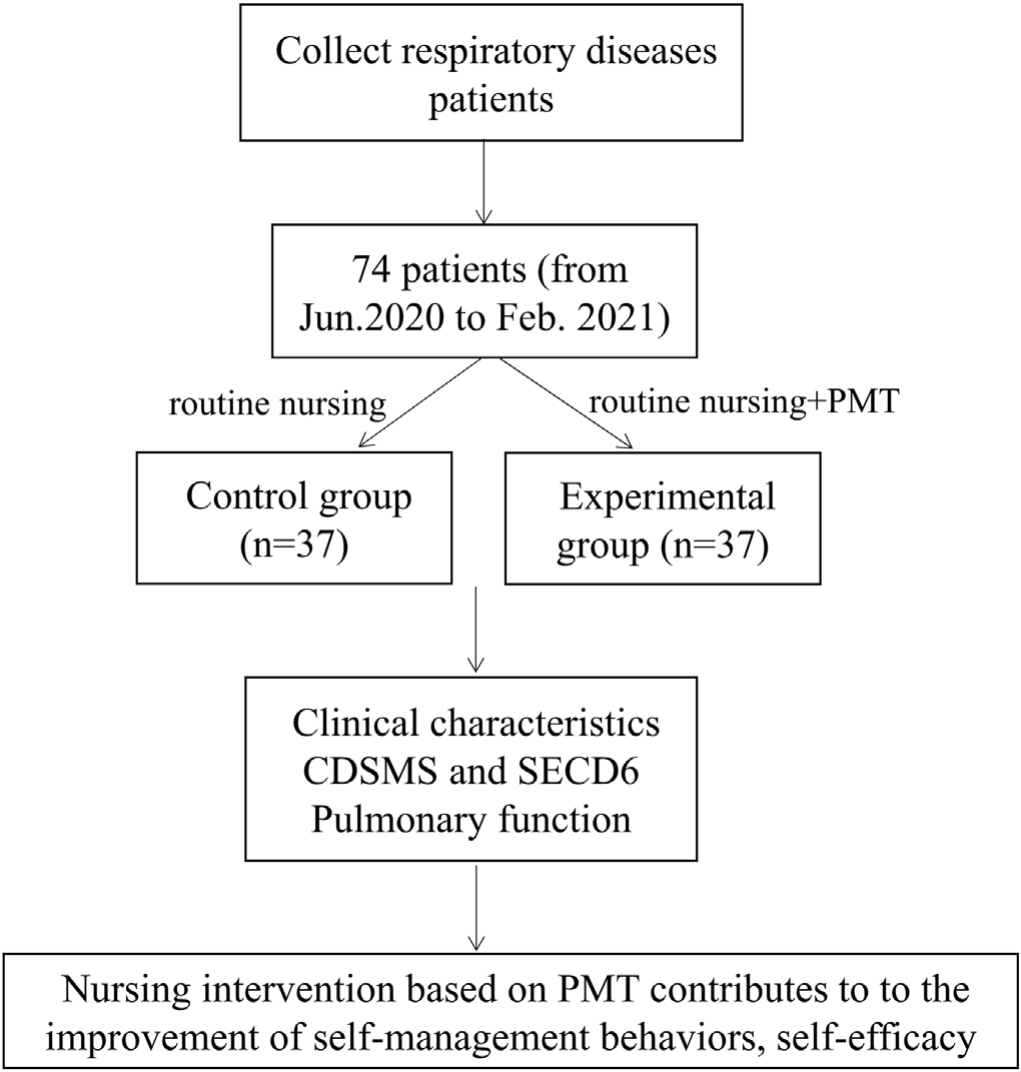
The flowchart of this study.

Therefore, a total of 74 patients with respiratory diseases treated in the respiratory department of the First People's Hospital of Huzhou, the First Affiliated Hospital of Huzhou University from June 2020 to February 2021 were selected as the research subjects. The patients were stratified into a control group (*n* = 37) and an experimental group (*n* = 37) by stratified random sampling method. Inclusion criteria: Patients (1) diagnosed with respiratory diseases, including COPD, asthma and bronchiectasis, after consulting respiratory physicians and receiving imaging examinations such as chest CT or bronchoscopy; (2) aged over 18 years; (3) with stable condition; and (4) with informed consent. Exclusion criteria: (1) Suspected or confirmed COVID‐19 patients; (2) patients with incomplete clinical data; (3) patients complicated with other systemic serious diseases; (4) patients who have language communication difficulties, do not understand and are unable to cooperate with doctors. This study was carried out with the approval of the Ethics Committee of our hospital, and the ethics number is 20220303. The flowchart of this study is illustrated in Figure 1.

### 2.2 Nursing methods

The control group received routine respiratory care, which consisted of three parts: admission education, hospitalization nursing and discharge guidance. The nursing guidance involved disease management, medication management, diet management and respiratory function exercise. After discharge, the patients were followed up once a week by telephone to guide and answer questions.

On top of that, the experimental group received PMT‐based nursing intervention. An intervention team was established, including one respiratory doctor, two respiratory nurses and one researcher. Intervention content and measures were formulated based on the 7 kernel variables of threat appraisal (severity, vulnerability, intrinsic rewards and extrinsic rewards) and coping appraisal (response efficacy, self‐efficacy and response cost), and the intervention measures were modified and perfected under the review and guidance of medical and nursing experts. After the formulation of the program, the members of the group were trained in PMT theory, enabling them to master the core elements of PMT. And the practice of the intervention program was guided. The intervention lasted for 2 months, and the forms of the intervention included group education lectures, individualized guidance, meeting of patients' families, experience exchange meeting and telephone follow‐up. The intervention program included the following measures:

1. One week after admission, group education lectures were arranged to teach respiratory disease‐related knowledge, complications and serious consequences, and pamphlets on respiratory disease knowledge were issued to improve patients' awareness in the form of text. The main purpose was to make patients aware of the consequences of inactive self‐management, increase their understanding of disease severity and vulnerability, and promote the transformation of their behaviors.
2. Two weeks after admission, group education lectures were given to teach respiratory disease and self‐management knowledge; meetings of patients' families were organized to encourage the families of patients to participate in the self‐management process of patients, in the view of reducing the intrinsic and extrinsic rewards of patients.
3. Three weeks after admission till discharge, experience exchange meetings were held to share experiences and motivate each other to increase patients' expectations for disease recovery. Throughout the hospital stay, individualized instruction was adopted to help patients to master correct self‐protection knowledge, self‐respiratory function exercise skills, healthy diet knowledge, and basic knowledge of the disease and medication. These instructions were given to increase patients' awareness of the benefits of healthy behaviors and strengthen patients' confidence in behavior improvement, which was conducive to enhancing patients' response efficacy and self‐efficacy.
4. After discharge, telephone follow‐up was conducted to pay attention to the psychological changes of patients, prevent patients from giving up disease management due to difficulties and timely give patients necessary psychological intervention, to help patients overcome the adverse factors affecting the rehabilitation of the disease and reduce the response cost.

### 2.3 Research tools

#### 2.3.1 General information questionnaire

Through consulting relevant literature and considering the actual situation of the project, the questionnaire was formulated by the researchers, and the main contents included general information about the study subjects and disease‐related information. The former included age, gender, height, weight, place of residence, marital status, educational level, occupation and income, whereas the latter included the type, course and complications of respiratory system diseases.

#### 2.3.2 Pulmonary function assessment

The levels of forced vital capacity (FVC), forced expiratory volume in 1 second (FEV1), peak expiratory flow (PEF) and FEV1/FVC were measured to evaluate pulmonary function.

#### 2.3.3 Chronic Disease Self‐Management Study Measures (CDSMS)

CDSMS was adopted to evaluate the effect of implementing a chronic disease self‐management program.^15^ This scale is mainly composed of three dimensions (15 items in total), namely exercise, cognitive symptom management practice and communication with doctors (Table S1). Each item is scored using a 4‐level scoring method, and the total score ranges from 15 to 60. The higher the score, the better the self‐management behavior of patients.

#### 2.3.4 Self‐Efficacy for Managing Chronic Diseases 6‐item Scale (SECD6)

SECD6 was employed to measure the level of self‐efficacy of patients.^16^ This scale consists of six items (Table S2), each of which is scored on a 10‐point scale from 1 (not confident at all) to 10 (completely confident), reflecting the self‐efficacy of patients with chronic diseases in terms of symptom management, role function, emotional control and communication with doctors. The total score ranges from 6 to 60, and the higher the score, the higher the level of self‐efficacy.

### 2.4 Data collection

Patient data were collected and first assessed before intervention in enrolled patients. Following 1 and 4 weeks of intervention, patients were assessed again. The researchers used a unified instruction language to explain the specific filling requirements of the scale to the subjects. Throughout the process, a unified explanatory language was used to answer any questions. For patients with low educational levels and difficulty in reading, the researchers expressed the items of the scale in a neutral tone and assisted them to fill in.

### 2.5 Statistical methods

Statistical analysis was performed using SPSS 25.0. Measurement data were expressed as mean ± standard deviation. Independent sample *t*‐test was used for measurement data fitting normal distribution, and rank sum test was used for measurement data that did not meet normal distribution. Enumeration data were expressed as frequency and percentage, and the chi‐square test or Fisher exact test was employed for them. Repeated measures analysis of variance was used to compare the outcomes of intervention at different periods. The test level was set as α = 0.05, and *p* < 0.05 indicated statistical significance.

## 3 RESULTS

### 3.1 Clinical characteristics of patients

In the control group, males accounted for 72.97% (27/37 cases), the average age was 74.27 ± 9.83 years, and the average course of the disease was 13.33 ± 9.93 years. The control group included 33 cases (89.19%) of COPD, three cases (8.11%) of bronchiectasis and four cases (10.81%) of asthma. In the experimental group, males accounted for 86.49% (32/37 cases), and the average age was 70.32 ± 7.12 years. Thirty‐seven cases (100.00%) were COPD, four cases (10.81%) were bronchiectasis, and two cases (5.41%) were asthma. The mean duration of the disease was 10.93 ± 8.45 years. There was one case with unknown course and comorbidities in the control group. Before the intervention, the results of the *t*‐test and chi‐square test showed no significant differences in baseline characteristics such as gender, BMI, education level, residence, occupation, income, marital status, smoking status, disease type, and course between the control group and the experimental group (*p* > 0.05), which indicated comparability, as shown in Table 1.

**TABLE 1:** Baseline characteristics of the two groups.

Characteristic	Control group (*n* = 37)	Experimental group (*n* = 37)	*p*‐Value
Age (years)	74.27 ± 9.83	70.32 ± 7.12	0.052
Gender, *n* (%)			0.247
Male	27 (72.97)	32 (86.49)	
Female	10 (27.03)	5 (13.51)	
BMI (kg/m^2^)	22.33 ± 3.70	20.87 ± 4.14	0.114
Place of residence, *n* (%)			0.482
Cities and towns	23 (62.16)	19 (51.35)	
Rural area	14 (37.84)	18 (48.65)	
Marital status, *n* (%)			0.564
Married	28 (75.68)	31 (83.78)	
Other (unmarried, divorced, widowed)	9 (24.32)	6 (16.22)	
Educational level, *n* (%)			0.227
Illiterate or semi‐illiterate	15 (67.57)	8 (21.62)	
Primary school	12 (32.43)	17 (45.95)	
Junior high school	8 (21.62)	7 (18.92)	
High school/technical secondary school	2 (5.41)	5 (13.51)	
College and above			
Occupation, *n* (%)			0.219
Worker	5 (13.51)	3 (8.11)	
Farmer	11 (29.73)	16 (43.24)	
Retired	18 (48.65)	18 (48.65)	
Other	3 (8.11)	0 (0)	
Per capita monthly household income (Yuan), *n* (%)			0.052
<1000	7 (18.92)	2 (5.41)	
1000–2000	21 (56.76)	25 (67.57)	
2000–3000	4 (10.81)	9 (24.32)	
>3000	5 (13.51)	1 (2.70)	
Inhabiting information, *n* (%)			0.493
Living with family (children and/or loved ones)	35 (94.59)	37 (100.00)	
Other (alone/unknown)	2 (5.41)	0 (0.00)	
Medical payment method, *n* (%)			0.157
Medical insurance	25 (67.57)	18 (48.65)	
Rural cooperative medical service	12 (32.43)	19 (51.35)	
Types of respiratory diseases, *n* (%)			
COPD	33 (89.19)	37 (100.00)	0.115
Bronchiectasis	3 (8.11)	4 (10.81)	1.000
Asthma	4 (10.81)	2 (5.41)	0.674
Smoking status, *n* (%)			0.126
Smoker	12 (32.43)	9 (24.32)	
Non‐smoker	18 (48.65)	13 (35.14)	
Ex‐smoker	7 (18.92)	15 (40.54)	
Course of disease (years)	10 (5.00, 20.00)	10 (5.00, 20.00)	0.258
Number of complications (number)	1 (1.00, 2.00)	1 (0.50, 2.00)	0.649

### 3.2 Chronic disease self‐management behaviors and self‐efficacy assessment

As shown in Table 2, before the intervention, the self‐management behavior and self‐efficacy scores of the experimental group had no statistically significant difference compared with the control group (*p* > 0.05), but at the first week and the fourth week of intervention, the scores of the experimental group were significantly higher than those of the control group (*p* < 0.0001). F‐statistical results showed that the self‐management behavior (*p* < 0.001) and self‐efficacy (*p* < 0.0001) of the two groups continued to increase during the intervention period, with changes more evident in the experimental group.

**TABLE 2:** Comparison of self‐management behaviors and self‐efficacy scores between two groups.

	Group	Before intervention	After 1 week of intervention	After 4 weeks of intervention	F (*p*‐value)
Self‐management behavior score	Control group	9.76 ± 4.57	11.84 ± 4.43	15.30 ± 4.50	82.679 (0.000)
	Experimental group	8.68 ± 4.20	16.08 ± 4.06	23.38 ± 3.97	275.389 (0.000)
T (*p*‐value)		1.060 (0.293)	−4.299 (0.000)	−8.185 (0.000)	
Self‐efficacy score	Control group	29.54 ± 5.10	32.41 ± 6.62	35.95 ± 8.21	34.711 (0.000)
	Experimental group	26.22 ± 5.60	38.65 ± 4.20	51.27 ± 4.36	667.232 (0.000)
T (*p*‐value)		2.670 (0.009)	−4.841 (0.000)	−10.027 (0.000)	

### 3.3 Pulmonary function assessment

As shown in Table 3, no significant differences were seen in pulmonary function indexes including PEF%, FEV1%, FVC% and FEV1/FVC% between the two groups before intervention (*p* > 0.05). After 1 week of intervention, there was no significant difference in pulmonary function between the experimental group and the control group (*p* > 0.05). Similarly, after 4 weeks of intervention, there was no significance in the pulmonary function indexes between the experimental group and the control group (*p* > 0.05).

**TABLE 3:** Comparison of pulmonary function indexes.

	Group	Before intervention	After 1 week of intervention	After 4 weeks of intervention	F (*p*‐value)
PEF predict (%)	Control group	55.74 ± 5.99	56.95 ± 5.94	60.26 ± 6.46	5.380 (0.006)
	Experimental group	54.84 ± 3.90	57.69 ± 3.78	61.08 ± 4.89	20.323 (0.000)
T (*p*‐value)		0.768 (0.445)	0.637 (0.526)	0.618 (0.539)	
FEV1 predict (%)	Control group	38.17 ± 7.84	44.48 ± 7.03	57.33 ± 6.67	68.136 (0.000)
	Experimental group	39.89 ± 7.72	45.62 ± 8.21	59.26 ± 9.63	50.023 (0.000)
T (*p*‐value)		0.949 (0.346)	0.641 (0.524)	1.002 (0.320)	
FVC predict (%)	Control group	47.46 ± 6.15	54.72 ± 6.16	65.89 ± 5.90	86.605 (0.000)
	Experimental group	44.83 ± 6.67	53.74 ± 5.11	62.73 ± 1.14	44.221 (0.000)
T (*p*‐value)		1.767 (0.082)	0.749 (0.457)	1.495 (0.141)	
FEV1/FVC (%)	Control group	62.75 ± 5.92	63.73 ± 5.40	68.32 ± 4.11	12.088 (0.000)
	Experimental group	65.94 ± 1.55	61.34 ± 6.31	70.53 ± 1.56	4.498 (0.013)
T (*p*‐value)		1.173 (0.247)	1.753 (0.084)	0.835 (0.406)	

## 4 DISCUSSION

The COVID‐19 outbreak has exerted a significant effect on the management of patients with chronic respiratory diseases, who are generally at high risk of infection during the COVID‐19 pandemic.^17^ Studies have shown that patients with chronic respiratory diseases are more likely than others to experience physical and mental health problems such as nasal discharge or congestion, chest congestion, fever and chills during the COVID‐19 pandemic.^18^ Therefore, prudent implementation of prevention and management strategies is essential for reducing the risk of COVID‐19 infection in patients with chronic respiratory diseases. Based on PMT, we implemented nursing interventions including health education, experience exchange and telephone follow‐up in patients with respiratory diseases to promote the rehabilitation of lung diseases and meet the demand for COVID‐19 infection prevention.

Clinical nursing usually adopts individualized self‐management to deal with various disease risks, and such self‐management plays an important role in the establishment of healthy behaviors.^19^ Self‐management refers to people's expectations, perceptions, confidence or beliefs about their ability to complete the actions required to achieve specific goals. Eroglu and Sabuncu^20^ implemented the interventions of education and telephone reminding in patients with type 2 diabetes, and as a result, the self‐management and self‐efficacy of patients were significantly improved, and their metabolic values were significantly reduced. A study on the impact of self‐management intervention on heart failure showed that self‐management intervention can consolidate heart failure‐related knowledge of patients, improve quality of life and reduce heart failure‐related hospitalization.^21^ Results of another clinical randomized controlled trial showed that providing disease‐related education for 61 COPD patients through an education‐based intervention program partially relieved the dyspnea of patients and significantly improved patients' self‐care management.^22^ Based on the above studies, it is worthy of the nursing staff's thinking and attention that how to improve the self‐behavior management ability of chronic disease patients and ultimately make them benefit from disease prevention through effective nursing management.

At present, there is no definitive consensus on whether patients with chronic respiratory disease are at increased risk of SARS‐CoV‐2 infection due to associated hospitalization,^23^ but clinical care during hospitalization is essential for preventing airborne transmission of viruses. PMT nursing practice shows a prominent effect on improving patients' self‐management ability, which enables patients to benefit from the long‐term disease management process. An information‐motivation‐behavior intervention program based on PMT helps to improve psychological resilience and quality of life in patients with type 2 diabetes while facilitating the reduction of blood glucose levels and depression scores.^24^ After 6 months of applying PMT in skin cancer prevention among elementary school students in rural areas of Fars, Iran, it helps to improve the effectiveness of elementary school students regarding skin cancer prevention.^25^ Khazaeian et al.^26^ found that after PMT training for female high school students in Zahedan, Iran, the intervention group receiving the training had higher scores in knowledge, theoretical structure and preventive behavior, indicating that PMT could effectively prevent osteoporosis. In this study, at weeks 1 and 4 after the PMT intervention, the self‐management behavior and self‐efficacy scores of the two groups continued to increase, but the change was more significant in the experimental group (*p* < 0.0001), which demonstrated that PMT‐based nursing intervention could effectively improve the quality of life of patients, thereby benefiting different types of patients. Furthermore, most of the current studies focus on the PMT‐based prediction of intentions and behaviors,^27^, ^28^, ^29^ whereas the preventive effect of nursing intervention on COVID‐19 in patients with chronic respiratory diseases remains unclear. In this study, the effect of PMT‐based nursing intervention was evaluated using CDSMS and SECD6 scales, and the pulmonary function‐related indicators PEF, FEV1 and FVC were measured. The results showed that after 1 and 4 weeks of intervention, pulmonary function indicators of the experimental group were slightly better than the control group, which was conducive to the recovery of patients' pulmonary function. To the best of our limited knowledge, this study is the first to apply PMT to the implementation of a clear nursing intervention program in patients with chronic respiratory disease during the COVID‐19 pandemic and has achieved satisfactory results. Nevertheless, the final number of patients included in this study is relatively small, and the nursing intervention time is short. Accordingly, the long‐term effect of PMT‐based nursing intervention needs to be further investigated. Besides, the role of PMT in predicting COVID‐19 prevention behaviors in patients with chronic respiratory disease also needs to be further clarified. In conclusion, PMT‐based nursing intervention in this paper has achieved good results in patients with chronic respiratory disease, which provides a reference for respiratory nursing during the period of the COVID‐19 epidemic.

## AUTHOR CONTRIBUTIONS

Jian Guan and Yingying Zhang contributed to the study design. Shan You conducted the literature search. Yujing Li acquired the data. Hongxing Zhao and Weiqin Bu wrote the article. Yanping Xie performed data analysis and drafted. Jian Guan revised the article. Yingying Zhang gave the final approval of the version to be submitted.

## CONFLICT OF INTEREST STATEMENT

The authors declare that they have no potential conflicts of interest.

## ETHICS STATEMENT

All methods were performed in accordance with the Declaration of Helsinki. The verbal consent was obtained and was approved by the ethics committee of the First People's Hospital of Huzhou, the First Affiliated Hospital of Huzhou University.

## Supporting information


**TABLE S1.** Chronic Disease Self‐Management Behaviors Scale.

**TABLE S2.** Self‐Efficacy for Managing Chronic Disease Scale.

## Data Availability

The data and materials in the current study are available from the corresponding author on reasonable request.
