# Supplementary material for: Application of protection motivation theory in epidemic prevention in patients with respiratory diseases under the COVID‐19 pandemic: A cross‐sectional study
Source: Clin Respir J. 2023 Sep 4;17(10):1058–66. doi: 10.1111/crj.13693 (PMC10542998; doi:10.1111/crj.13693)
Supplement: Supplementary file 1 — TABLE S1. Chronic Disease Self‐Management Behaviors Scale. [file CRJ-17-1058-s001.docx]

**Supplementary Table 1**

**Chronic Disease Self-Management Behaviors Scale**

**Exercise：How much time did you spend on the following activities over the past week? (Please tick under each question)**

|  | Null | <30 min/week | 30-59 min/week | 1-3 h/week | >3 h/week |
| --- | --- | --- | --- | --- | --- |
| 1. Fitness exercises (continuous movements, lifting dumbbell, etc) |  |  |  |  |  |
| 2. Walk |  |  |  |  |  |
| 3. Swim |  |  |  |  |  |
| 4. Bicycle riding |  |  |  |  |  |
| 5. Exercise with exercise equipment (treadmill, trampoline, etc) |  |  |  |  |  |
| 6. Other aerobic exercises (running, playing table tennis, etc) |  |  |  |  |  |

**Cognitive symptom management: When you feel sad, sad, or other uncomfortable situation, how do you deal with it? (Please tick under each question)**

|  | Null | Occasionally | Sometimes | Frequently | Very often | Always |
| --- | --- | --- | --- | --- | --- | --- |
| 1. Ignore the feeling |  |  |  |  |  |  |
| 2. Think of it as a common sensation, such as warmth, numbness, etc |  |  |  |  |  |  |
| 3. Dissolve by other means, such as listening to music |  |  |  |  |  |  |
| 4. Muscle relaxation exercises |  |  |  |  |  |  |
| 5. Self-adjustment, such as visualizing yourself to a comfortable place |  |  |  |  |  |  |
| 6. To be optimistic |  |  |  |  |  |  |

**Communication with your doctor: When you are on outpatient appointment, what will you often do? (Please tick under each question)**

|  | Null | Occasionally | Sometimes | Frequently | Very often | Always |
| --- | --- | --- | --- | --- | --- | --- |
| 1. Think about the questions ahead of time and make a list |  |  |  |  |  |  |
| 2. Consult about unclear aspects of the disease |  |  |  |  |  |  |
| 3. Discuss personal questions about illness with the doctor |  |  |  |  |  |  |
