# Supplementary material for: Application of protection motivation theory in epidemic prevention in patients with respiratory diseases under the COVID‐19 pandemic: A cross‐sectional study
Source: Clin Respir J. 2023 Sep 4;17(10):1058–66. doi: 10.1111/crj.13693 (PMC10542998; doi:10.1111/crj.13693)
Supplement: Supplementary file 2 — TABLE S2. Self‐Efficacy for Managing Chronic Disease Scale. [file CRJ-17-1058-s002.docx]

**Supplementary Table 2**

**Self-Efficacy for Managing Chronic Disease Scale**

Guide: The following questions are designed to investigate your confidence in doing daily matters. On a scale of 1 to 10, with 1 being “no confidence” and 10 being “absolute confidence”, choose a number that truly reflects your confidence in the following items.

No confidence Absoulute conficence

0 1 2 3 4 5 6 7 8 9 10

| Item | Score |
| --- | --- |
| 1. How confident are you that you want to do something, but are hampered by fatigue from illness? |  |
| 2. How confident are you that you want to do something, but are hampered by body unwell or pain from illness? |  |
| 3. How confident are you that you want to do something, but are hampered by down in the dumps from illness? |  |
| 4. How confident are you that you want to do something, but are hampered by other symptoms or health problems from illness? |  |
| 5. How confident are you that you can take different actions to manage your health to reduce doctor visits? |  |
| 6. How confident are you that you can take any actions other than medication to reduce the impact of the disease on your daily life? |  |
